# A randomized controlled trial of high volume simethicone to improve visualization during capsule endoscopy

**DOI:** 10.1371/journal.pone.0249490

**Published:** 2021-04-01

**Authors:** Michael Sey, Brian Yan, Cassandra McDonald, Dan Segal, Joshua Friedland, Klajdi Puka, Vipul Jairath

**Affiliations:** 1 Division of Gastroenterology, Department of Medicine, Western University, London, Ontario, Canada; 2 Program for Experimental Medicine, Western University, London, Ontario, Canada; 3 Lawson Health Research Institute, London Health Sciences Centre, London, Ontario, Canada; 4 Niagara Health System, McMaster University, Hamilton, Ontario, Canada; 5 Department of Medicine, University of Toronto, Toronto, Ontario, Canada; 6 Department of Epidemiology and Biostatistics, Western University, London, Ontario, Canada; National Cancer Institute, UNITED STATES

## Abstract

**Background:**

The optimal dose of simethicone before capsule endoscopy is unknown. Prior studies have reported inconsistent cleansing, with some showing improved visualization only in the proximal small intestine. We hypothesized a higher volume of simethicone may improve cleansing and diagnostic yield, especially in the distal small bowel.

**Methods:**

A phase III randomized controlled trial was conducted comparing high volume (1125 mg simethicone in 750 ml water) versus standard volume (300 mg simethicone in 200 ml water) solutions, both at 1.5 mg/ml. The primary outcome was adequate bowel preparation, defined as a KOrea-CanaDA (KODA) score >2.25, overall and stratified by the proximal and distal half of the small bowel. Secondary outcomes included mean KODA score, diagnostic yield, completion rate, and adverse events. All analyses were intention-to-treat.

**Results:**

A total of 167 patients were randomized (mean (SD) age 58.7 (15.7), 54% female) and the most common indication was obscure gastrointestinal bleeding (71.7%). Adequate cleansing was achieved in 39 (50%) patients in the high volume group and in 39 (48%) patients in the standard volume group (RR 1.04, 95% CI 0.76–1.43, p = 0.82), with no differences observed in the proximal half (71% vs 64%, p = 0.40) or the distal half -of the small bowel (36% vs. 37%, p = 0.88). There was no differences in the mean (SD) KODA score (2.20 (0.41) vs. 2.18 (0.44), p = 0.73), diagnostic yields (53% vs. 56%, p = 0.71), or completion rates (both 95%). One adverse event, nausea, occurred in the control group.

**Conclusion:**

High volume simethicone does not improve visualization during capsule endoscopy.

**Clinical trial registration:**

**Clinical trial:** NCT02334631.

## Introduction

Since its invention at the turn of the millennium, capsule endoscopy has revolutionized small bowel imaging [1–3]. Despite this, diagnostic yields are still suboptimal. In a meta-analysis involving 15,074 patients with obscure gastrointestinal bleeding, capsule endoscopy was diagnostic in only 59% of cases [4]. There are several reasons for this. First, capsule endoscopy lacks air insufflation and cannot distend the lumen to permit optimal visualization. Second, the capsule cannot be controlled in a manner similar to conventional endoscopy, limiting visualization of portions of the small bowel. Third, bile, intestinal secretions, and bubbles cannot be washed and suctioned as it can during conventional endoscopy. Of these factors, the third is the only which can potentially be improved upon by clinicians, since the first two require changes in the engineering and design of the capsule. Accordingly, clinical research in the field has focused on improving bowel preparation before capsule endoscopy to improve visualization. Two meta-analyses of randomized controlled trials have reported improved visualization quality with the use of purgative laxatives and an anti-foaming agent, usually in the form of simethicone [5,6]. As a result, the latest North American and European clinical guidelines support the use of both before capsule endoscopy [2,3].

**Fig 1:**
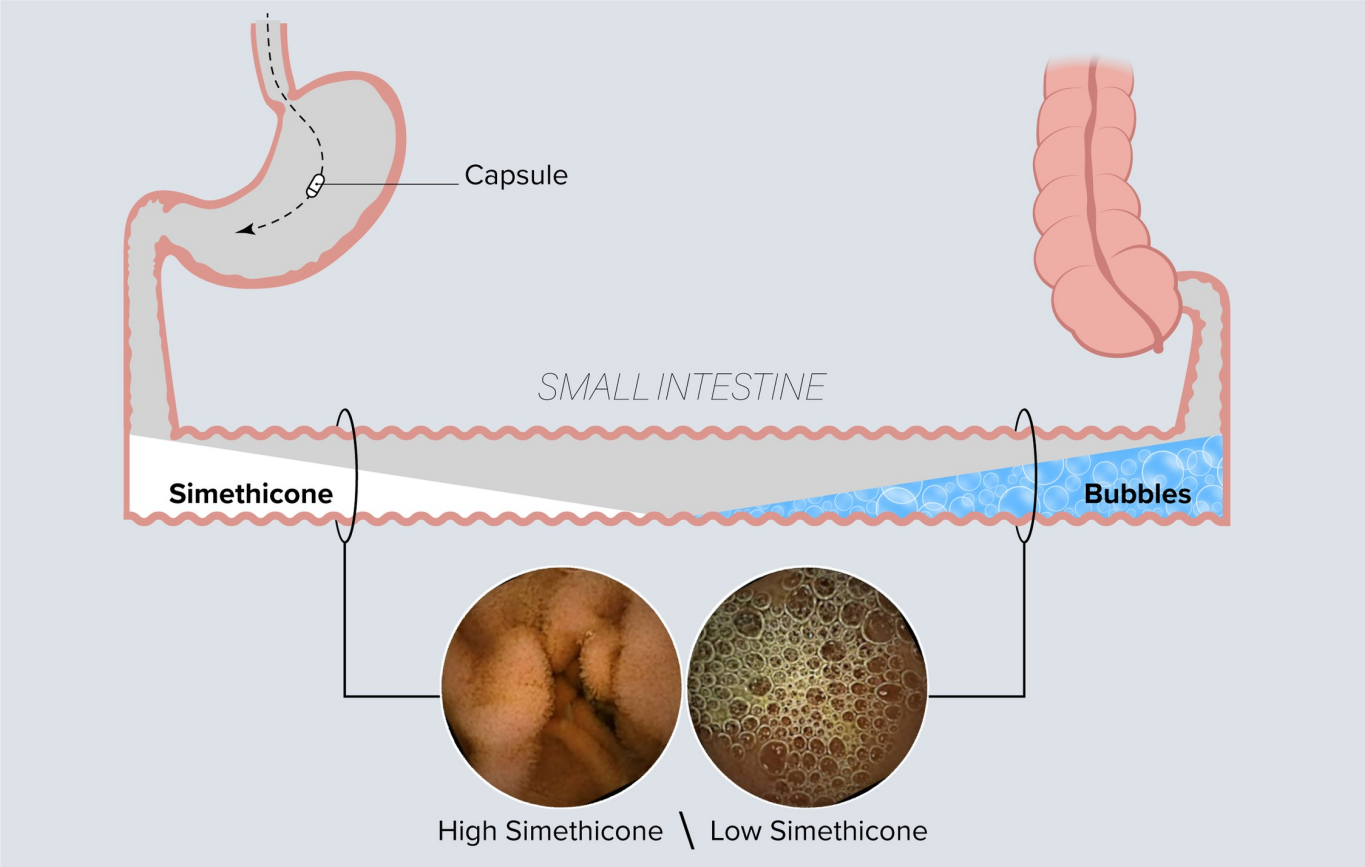
Poor cleansing of the distal small bowel due to inadequate volume of simethicone solution used.

Despite this, there is uncertainty as to the optimal dose of simethicone to use. Simethicone, a non-absorbable surfactant that reduces surface tension of bubbles leading to their collapse and dispersion, is used as an adjunct to purgative laxatives, such as polyethylene glycol with electrolyte lavage solution (PEG-ELS) [7]. Mechanistically, there may be synergy between the two in the setting of capsule endoscopy. Although PEG-ELS purges the small intestine of luminal contents, it does not remove bubbles and may paradoxically contribute to their formation [8,9]. As such, there is biologic plausibility to support the routine use of simethicone as an adjunct to disperse bubbles before capsule endoscopy. However, the beneficial effects of simethicone in clinical trials have been inconsistent, with some studies reporting improved visualization [10–12], some reporting no improvement [13,14], and some reporting improvement only in the proximal small intestine [15,16]. The volume of simethicone solution used in each trial was not standardized, although up to 200 ml were used in two studies [10,11,13–16]. Given the length of the small intestine, measuring on average over 6 meters in length [17], the inconsistent cleansing may be the result of an inadequate volume of simethicone solution being used in prior studies (Fig 1). We conducted a randomized controlled trial to test whether high volume simethicone would improve visualization of the total small intestine and in particular, the distal small intestine, compared to standard volume.

## Material and methods

### Study design and setting

We conducted a phase III randomized controlled trial at the London Health Sciences Centre-Victoria Hospital, a tertiary care centre affiliated with Western University. Our Small Intestinal Endoscopy Program serves the entire Southwest region of the province of Ontario, Canada, and consists of 1.4 million inhabitants and a geographic area of 33,673 square kilometers [18]. The trial was conducted according to Good Clinical Practice guidelines [19], approved by the Western University Research Ethics Board (HSREB #106269), and registered at www.clinicaltrials.gov (NCT02334631). Due to the use of a supratherapeutic dose of simethicone, registration and authorization as a phase III clinical trial was obtained from Health Canada prior to study initiation (HC6-24-c 181580). The study received REB approval on December 21, 2016, recruited the first participant on February 2, 2017, and enrolled the last participant on July 24, 2019.

### Patients

All patients undergoing outpatient small intestinal capsule endoscopy were screened for participation in the trial by research personnel. Patients who had a contraindication to capsule endoscopy, underwent endoscopic insertion of the capsule, had a capsule study as an inpatient for active small intestinal bleeding, on a fluid restriction, or who felt they were unable to drink up to 900 ml of fluid within 10 minutes prior to the capsule were excluded.

### Intervention

There is no widely accepted or recommended standard volume of simethicone used before capsule endoscopy. Thus, we chose the volume in the control arm based on a literature review, which revealed that most existing clinical trials generally used 0.5 to 200 ml, often at a concentration of 1.5 mg/ml [10,11,13–16,20] Given the large reservoir capacity of the stomach and the considerable length of the small intestine, we selected a simethicone solution of 200 ml at 1.5 mg/ml as the control. In *ex vivo* testing, we found that concentrations >1.5 mg/ml produced an overly cloudy and opaque solution that may paradoxically reduce visualization. For the experimental arm, we selected a simethicone solution of 750 ml at 1.5 mg/ml. This was based on pilot data from 15 patients, which demonstrated the volume to be palatable and well tolerated. All simethicone solutions were prepared at randomization by diluting simethicone drops (Ovol® Drops, 40 mg/ml, Church & Dwight Canada Corp) with water until the desired volume (200 ml vs. 750 ml) and concentration (1.5 mg/ml) was reached.

### Randomization procedures

Randomization was performed in unequal sized blocks (size 2, 4, 6) in a parallel arm fashion with 1:1 allocation between the experimental and control arms by the research personnel. Research Electronic Data Capture (REDCap), hosted at Western University, was utilized for web based randomization and data collection. The randomization schema was concealed electronically by REDCap and the randomization allocation was available one subject at a time and only after completion of study enrollment. There were no concerns for the integrity of allocation concealment, which was also supported by the Berger-Exner test (p = 0.29 (treatment), and p = 0.28 (control)) [21,22].

#### Blinding

Patient blinding was difficult since the two treatment arms involved ingesting different volumes of solution (i.e. 200 ml vs. 750 ml). This created an opportunity for unmasking as patients may deduce their treatment arm based on the weight of the cup and the amount ingested. Equalization of volume using water could not be used due to dilution. As a result, de facto masking [23] was used whereby patients were not informed of the volumes being compared until after the study. Thus, even if patients were able to estimate the solution volume based on weight or amount ingested, they would remain blinded to randomization allocation since they would not know the volumes being compared. To make volume estimation more difficult, identical tall white plastic narrow mouth bottles that are difficult to see into were used. To meet the requirements of our REB, patients were informed beforehand that they would be asked to drink a simethicone solution between 1 to 900 ml in volume. Upon completion of the study, they were given a letter informing them of the volumes in the two treatment arms and their randomization allocation.

All physicians and outcome assessors were blinded to randomization allocation. To ensure this, research activities, including screening, enrollment, consent, baseline data collection, randomization, and medication administration, were conducted solely by research personnel.

### Capsule endoscopy procedures

All patients followed a clear fluid diet the day before the procedure, ingested 2L PEG-ELS between 8–10 PM (PegLyte®, PendoPharm Inc., Montreal, Canada), and began fasting at midnight. Patients arrived at 6:30 AM to the endoscopy unit, were screened, and offered participation in the trial. Those who enrolled were randomized and given either the control or experimental volume of simethicone solution and asked to drink it over 5 minutes. Bottles were collected afterwards to assess adherence. The patients were then sent back to the waiting room for 30 minutes to allow the simethicone solution to enter the small intestine before ingesting the capsule (PillCam™ SB3, Medtronic, Yoqneam, Israel). Patients were permitted to have clear fluids in 2 hours and a light meal in 4 hours. The recorder was returned to the endoscopy unit at 5 PM. Live images on the recorder were then assessed by a nurse and if the capsule was in the colon, the recorder was retrieved and the images downloaded. If the live images showed the capsule to still be in the small intestine, the patient was asked to keep the recorder overnight and return it to the endoscopy unit the following morning instead. All capsules were read in RAPID 8.0 (Medtronic, Yoqneam, Israel).

### Outcomes

Small bowel preparation quality was assessed using the KOrea-CanaDA (KODA) score, which we previously validated for use with capsule endoscopy with almost perfect inter-rater (ICC 0.81, 95% CI 0.70–0.87) and intra-rater reliabilities (ICC 0.92, 95%CI 0.87–0.94) among 20 readers of varying occupational backgrounds [24]. This score was utilized as there are no other validated bowel preparation score for the small intestine. In brief, the KODA score assesses bowel preparation quality on two domains using ratings between 0 to 3: percentage of visualized mucosa seen (3: >75%, 2: 50–75%, 1: 25–49%, 0: <25%) and percentage of view obstructed by bubbles, bile, or secretions (3: <5%, 2: 5–25%, 1: 26–50%; 0: >50%). The total score is a mean of the two sub-scores. Capsule studies were assessed by dividing the small bowel images into 5 minute segments and assessing the first image within each segment. The reliability of this approach was previously established, with one study reporting almost perfect correlation (ICC = 0.82) between sampling images every 5 minutes and sampling every image within the first 2 minutes of every 5 minute segment (e.g. 11,520 images sampled and scored in a 4-hour video) [25]. All capsule videos were read blindly in duplicate using RAPID 8.0 by an experienced capsule reader (MS who has read >500 capsules) and a research assistant (CM) with no capsule experience but who completed a web based training module for the KODA score (https://www.schulich.uwo.ca/gastroenterology/research/research_tools.html).

The primary outcome was adequate bowel preparation, defined as a KODA score >2.25 [24]. Secondary outcomes were: 1) Mean KODA score, for the total, proximal half, and distal half of the small intestine; 2) Diagnostic yield, defined as an angioectasia, polyp, tumor/mass, ulcer, Crohn’s disease, or stricture (small non-specific red spots of questionable significance were not considered diagnostic); 3) Study completion rate, defined as the capsule reaching the cecum; 4) Gastric transit time, defined as the time interval between the first gastric and first duodenal image; 5) Small intestinal transit time, defined as the time interval between the first duodenal and first cecal image. Given the large volume of simethicone solution ingested in the experimental arm, secondary outcomes #3–5 were included to ensure completion rates and transit times were not affected.

Adverse events were assessed on the procedure day and at day 7 by research personnel (telephone follow-up).

### Statistical analysis

Assuming an 80% bowel preparation adequacy rate in the experimental arm (87% from pilot data in 15 patients who took this regimen, Table in S1 Table), 60% in the control arm (53% from pilot data in 15 patients who took this regimen, Table in S1 Table), power of 0.80, and a two-sided 5%-level Peason’s *χ*^2^ test, 164 participants (82 per group) were needed in the study.

Analyses were completed using R 3.6.1. Mean and standard deviation (SD) were used to describe continuous variables, and proportions and percentages were used to describe categorical variables. All analyses were completed using intention to treat principle. The average KODA score between the two blinded readers was used. In comparing the two randomized groups on binary variables, risk ratios (RR) with 95% Wald Confidence Intervals (CI) were computed, as well as Fishers’ exact test. In comparing the two randomized groups on continuous variables, Mann-Whitney U test were used. Results were similar when chi-square and t-tests were used (results not shown).

## Results

### Patient characteristics

**Fig 2:**
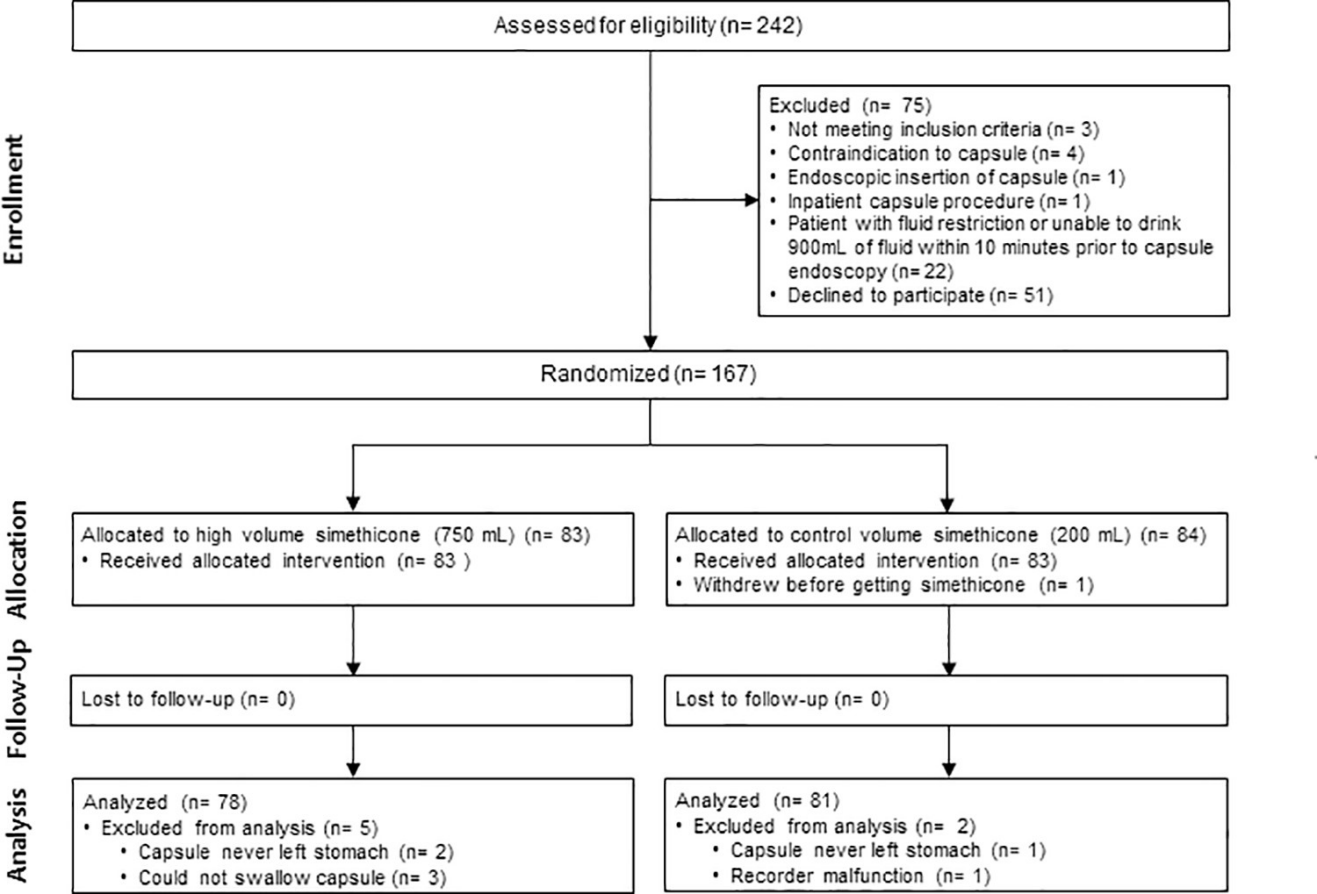
CONSORT flow diagram [26].

Between February 2017 and July 2019, a total of 242 patients undergoing outpatient capsule endoscopy were screened, of which 167 were enrolled and randomized, 83 to high volume and 84 to standard volume simethicone (Fig 2). Three patients in the high volume group could not swallow the capsule and did not undergo the procedure. The capsule in three patients never left the stomach (2 in high volume and 1 in standard volume simethicone group) and the recorder malfunctioned in one patient with no images captured.

Baseline characteristics between the two groups were similar (Table 1). On close examination of the magnitude of any differences between groups, while considering the prognostic strength of the variables on study outcomes, Table 1 shows that the two groups are similar across all baseline characteristics. The most common indication was obscure gastrointestinal bleeding, followed by Crohn’s disease. Among those randomized to the high volume group, 79/83 (95.2%) were able to drink all 750 ml of simethicone solution. For those who could not finish the high volume solution, all drank at least 500 ml (one drank 660 ml, one drank 560 ml, and two drank 500 ml). Among patients randomized to the standard volume group, 83/84 (98.8%) consumed 100% of the simethicone solution (200 ml). One subject in the standard volume group withdrew consent before receiving the study intervention.

**Table 1:** Baseline characteristics.

Baseline characteristics	High volume group	Standard volume group	p-value
Age-mean (SD)	57.94 (15.83)	59.48 (15.53)	.52
Sex, female-no. (%)	40 (51.3)	46 (56.8)	.44
Indication for capsule endoscopy-no. (%)			.54
Obscure, overt GI bleed	23 (29.5)	20 (24.7)	
Obscure, occult GI bleed	34 (43.6)	44 (54.3)	
Suspected/established Crohn’s disease	14 (17.9)	10 (12.3)	
Suspected polyp/tumor	7 (9.0)	7 (8.6)	
Previous Small Bowel Investigation-no. (%)			
Small bowel follow through	4 (5.1)	5 (6.2)	1.0
CT abdomen	33 (39.8)	23 (28.4)	.07
CT enterography	14 (17.9)	15 (18.5)	1.0
MR enterography	6 (7.7)	8 (9.5)	.78
RBC Scan	4 (4.8)	6 (7.1)	.75
Meckel’s Scan	4 (4.8)	4 (4.8)	1.0
Bowel Ultrasound	1 (1.3)	0 (0.0)	.49
PUSH Enteroscopy	5 (6.0)	5 (6.0)	1.0
Capsule endoscopy	7 (9.0)	10 (12.3)	.61
Single/double balloon enteroscopy	10 (12.8)	5 (6.2)	.18
Other	3 (3.6)	2 (2.4)	.68
Motility Impairing Medication Usage-no. (%)			
Opioid	9 (11.5)	5 (6.2)	.27
Anticholinergic	2 (2.6)	2 (2.5)	1.0
Dopaminergic medication	0 (0.0)	2 (2.5)	.50
Calcium channel blockers	11 (14.1)	12 (14.8)	1.0
Iron pills	1 (1.3)	8 (9.9)	1.0
Other	2 (2.6)	3 (3.7)	1.0
None	58 (74.4)	55 (67.9)	.39

### Bowel cleanliness and diagnostic rate

Adequate bowel preparation was achieved in 39 (50%) patients randomized to high volume simethicone and 39 (48%) patients randomized to standard volume (RR 1.04, 95% CI 0.76–1.43, p = 0.87). No difference in adequate bowel preparation was observed when assessment was limited to the proximal half (71% vs. 64%, RR 1.10, 95% CI 0.88–1.36, p = 0.40) or the distal half of the small intestine (36% vs. 37%, RR 0.97, 95% CI 0.64–1.46, p = 0.10) when comparing high volume and standard volume simethicone, respectively. The mean (SD) KODA score in the high volume group was 2.20 (0.41) and the mean (SD) KODA score in the standard volume group was 2.18 (0.44). No differences were observed in the mean KODA score for the proximal half or the distal half of the small bowel (Table 2).

**Table 2:** KODA scores for high volume vs. control volume simethicone.

	KODA Score, mean (SD)					
	Overall	p-value	% visualized mucosa sub-score	p-value	% view obstructed sub-score	p-value
**Total Small Intestine**						
High volume group	2.20 (0.41)	0.74	2.45 (0.38)	0.81	1.96 (0.44)	0.63
Standard volume group	2.18 (0.44)		2.43 (0.39)		1.92 (0.49)	
**Proximal Half of Small Intestine**						
High volume group	2.38 (0.43)	0.51	2.62 (0.38)	0.36	2.15 (0.50)	0.62
Standard volume group	2.34 (0.43)		2.57 (0.36)		2.11 (0.51)	
**Distal Half of Small Intestine**						
High volume group	2.03 (0.57)	0.92	2.28 (0.54)	0.90	1.77 (0.62)	0.79
Standard volume group	2.02 (0.57)		2.29 (0.52)		1.74 (0.63)	

The KODA scores are stratified by overall score, % visualized mucosa sub-score, and % view obstructed sub-score.

The capsule study was diagnostic in 53% of patients in the high volume group compared to 56% in the standard volume group (RR 0.95, 95% CI 0.71–1.26, p = 0.75) (Table 3).

**Table 3:** Findings on capsule study.

	High volume group n (%)	Standard volume group n (%)
Normal or non-specific red spots	37 (47%)	36 (44%)
Angioectasia	25 (32%)	32 (40%)
Ulcer(s)	2 (3%)	7 (9%)
Mass	2 (3%)	2 (3%)
Polyp	3 (4%)	1 (1%)
Stricture	1 (1%)	2 (3%)
Frank blood	5 (6%)	0 (0%)
Other	3 (4%)	1 (1%)

### Completion rate and transit times

The study completion rate was 95% in both arms of the study (RR 1.0, 95% CI 0.93–1.07, p = 1.0). There were no significant differences in the mean (SD) gastric transit time (30.4 (32.4) vs. 29.1 (36.8) minutes, p = 0.61) or the mean (SD) small intestinal transit time (225.7 (94.6) vs. 222.1 (101.6) minutes, p = 0.99) between the high volume and standard volume simethicone groups.

### Adverse events

There was one adverse event, nausea, reported in the standard group that resolved spontaneously. There were no other adverse events and no serious adverse events.

## Discussion

The latest North American and European guidelines recommend the use of an anti-foaming agent before capsule endoscopy based on two meta-analyses showing an overall benefit [2,3,5,6]. However, the optimal dose is unknown [3], with studies reporting either improved cleansing [10–12], no improvement [13,14], or improvement limited to the proximal small intestine [15,16]. Given the large pooling reservoir of the stomach and the long length of the small intestine, we hypothesized a larger volume of simethicone than previously used may produce more consistent cleansing, particularly in the distal half of the small intestine. In keeping with this hypothesis, our results showed poorer bowel preparation quality in the distal half compared to the proximal half of the small bowel (KODA score 2.02 vs. 2.36, p<0.001). However, despite giving nearly quadruple the volume of simethicone used in prior trials [10,11,13,15,16], we failed to detect any improvement in the visualization quality as measured by a validated small intestinal bowel preparation scale. Furthermore, there was no difference in bowel preparation adequacy in either the proximal or distal half of the small intestine. There are two possible explanations for these findings. First, there may be a ceiling effect whereby additional volume of simethicone may not yield any further improvement. Second, the volume used may be inadequate to produce a meaningful difference and even larger volumes are required. However, given we already nearly quadrupled the volume of what is typically used, we feel this is less likely. Furthermore, tolerability would become an issue if larger volumes are used. Thus, our results do not support the use of high volume simethicone before capsule endoscopy.

The primary strength of this study was the rigor in the design, including use of randomization, de facto masking, and centralized reading where adjudicators were blinded to treatment allocation and clinical information. In addition, we used a validated outcome measure with almost perfect inter and intra-rater reliabilities [24]. In contrast, prior studies [10,11,13–16] used un-validated and often *ad hoc* bowel preparation scales, which compromise their validity given their outcomes may change if assessed by a different individual (e.g. inter-rater reliability) or even by the same individual on a different day (e.g. intra-rater reliability). Lastly, our study was well powered to detect a difference, if it existed, between the experimental and control arms. Studies preceding ours were much smaller [10,11,13,14,16] or included too many treatment arms, resulting in a small sample size within each arm [15,27].

There are three limitations in the study that should be considered. First, the study did not include a placebo arm. However, given two meta-analyses [5,6] had already been published supporting the use of simethicone when the study was conceived, later supported by two international guidelines [2,3], we felt it unethical to include a placebo arm in our trial. Second, the study was not double blinded due to the inability to completely blind patients as a result of the different volumes of simethicone solutions ingested. However, we used de facto masking to prevent patients from deducing whether they were in the active or control arm by only revealing the volumes being compared upon completion of the study. Furthermore, we do not expect that knowledge of the volumes being compared, even if known, would have affected visual quality scores, which were adjudicated by blinded outcome assessors. Third, the study was limited to a single centre. However, given the large sample size and diversity of cases, we feel the results are still generalizable to other capsule programs.

In conclusion, high volume simethicone (750 ml, 1.5 mg/ml) is not superior to standard volume simethicone (200 ml, 1.5 mg/ml) and its use cannot be recommended before capsule endoscopy.

## Supporting information

S1 Checklist CONSORT 2010 checklist. (DOCX)

S1 Table Pilot data used to inform the sample size calculation. (DOCX)

S1 Text Study proposal. (DOCX)
