# Supplementary material for: A randomized controlled trial of high volume simethicone to improve visualization during capsule endoscopy
Source: PLoS One. 2021 Apr 1;16(4):e0249490. doi: 10.1371/journal.pone.0249490 (PMC8016230; doi:10.1371/journal.pone.0249490)
Supplement: S1 Table — (DOCX) [file pone.0249490.s002.docx]

**SUPPLEMENT**

**S1 Table. Pilot data used to inform the sample size calculation**

|  | **Standard Volume Simethicone**  **(n = 15)** | **High Volume**  **Simethicone**  **(n = 15)** | **Odds Ratio (95% CI)** | **p-value** |
| --- | --- | --- | --- | --- |
| Gastric transit time-mean (SD) | 21.9 (30.8) | 40.9 (31.8) | - | 0.11 |
| Small intestine transit time-mean (SD) | 251.0 (81.5) | 214.9 (119.3) | - | 0.34 |
| Incomplete study-no (%)^a^ | 0 (0%) | 1 (7%) | - | 0.31 |
| Diagnostic rate^b^-no (%)  Angioectasia  Small intestinal ulceration | 12 (80%)  10 (67%)  2 (13%) | 7 (47%)  6 (40%)  1 (7%) | 0.22 (0.04-1.11)  0.48 (0.09-2.52)  0.60 (0.05-7.63) | 0.06 |
| Visualized Mucosa Score-mean (SD)  Proximal half  Distal half  Entire small intestine | 2.53 (0.37)  2.28 (0.53)  2.45 (0.37) | 2.69 (0.33)  2.48 (0.24)  2.64 (0.18) | -  -  - | 0.21  0.19  0.08 |
| Degree of Obstruction Score-mean (SD)  Proximal half  Distal half  Entire small intestine | 2.17 (0.40)  1.90 (0.59)  2.09 (0.39) | 2.29 (0.48)  2.07 (0.36)  2.24 (0.32) | -  -  - | 0.47  0.34  0.25 |
| Visualization Quality Score-mean (SD)  Proximal half  Distal half  Entire small intestine | 2.35 (0.37)  2.09 (0.56)  2.23 (0.45) | 2.49 (0.40)  2.28 (0.27)  2.44 (0.24) | -  -  - | 0.32  0.25  0.13 |
| Adequate bowel preparation^c^ | 8 (53%) | 13 (87%) | 5.69 (0.94-34.5) | 0.05 |

^a^Denotes failure to reach the cecum prior to the end of the study

^b^Excludes small non-specific red spots

^c^Defined as visualization quality score for the entire small intestine ≥ 2.25.
