# Supplementary material for: A randomized controlled trial of high volume simethicone to improve visualization during capsule endoscopy
Source: PLoS One. 2021 Apr 1;16(4):e0249490. doi: 10.1371/journal.pone.0249490 (PMC8016230; doi:10.1371/journal.pone.0249490)
Supplement: S1 Text — (DOCX) [file pone.0249490.s003.docx]

**TITLE:** A double blind randomized controlled trial of high volume simethicone to improve visualization during capsule endoscopy

**VERSION #:** V1.21.2017

**STUDY SPONSOR/PI**

Michael Sai Lai Sey MD FRCPC

Assistant Professor of Medicine

Department of Medicine, Division of Gastroenterology

Western University

London Health Sciences Centre

800 Commissioners Rd. E.

London, ON, Canada

N6A 5W9

Tel: 519-667-6582

Fax: 519-667-6820

E-mail: [msey2@uwo.ca](mailto:msey2@uwo.ca)

**LAY SUMMARY**

Capsule endoscopy (CE) involves swallowing a pill with a tiny camera built into it to examine the small intestine. Unfortunately, air bubbles inside the bowel can block the view of the camera (Figure 1) and lead to missed diagnoses, such as cancers, polyps, abnormal blood vessels, and ulcers. Simethicone is a liquid medication swallowed 30 minutes before CE that stays inside the bowels to get rid of air bubbles. Prior studies have tried using this medication with mixed success. We believe that simethicone is likely effective but is not being given in a high enough volume to clean the entire small intestine. In our study, we will randomize people to either the standard amount of simethicone (200 ml) or high volume simethicone (750 ml) to determine if the latter will remove air bubbles better and lead to improved views of the small intestine and more diagnoses. We have already conducted a mini pilot study that showed a strong trend supporting our hypothesis that the high volume simethicone works better. However, confirmation of these findings by a rigorous clinical trial is needed.

**BACKGROUND, RATIONALE, & PILOT DATA**

Introduction

Capsule endoscopy (CE) involves the ingestion of a minature camera built into a small capsule to visualize the gastrointestinal tract.([1](#_ENREF_1)) Since its invention in the year 2000, CE has revolutionized small bowel imaging.([2-5](#_ENREF_2)) Prior to its development, conventional endoscopy could not examine the vast majority of the small intestine, leading to missed diagnoses in some patients and the need for surgery in others. Over the past decade, CE has become the diagnostic test of choice for many small intestinal diseases, including obscure gastrointestinal bleeding, isolated small bowel Crohn’s disease, refractory Celiac disease, and small bowel polyps and tumors with diagnostic rates exceeding radiologic studies and matched only by intraoperative enteroscopy.([1](#_ENREF_1), [4](#_ENREF_4), [6-20](#_ENREF_6)) Despite these merits, CE is limited by the need for a clean intestinal lumen. Unlike gastroscopy or colonoscopy, CE does not have the capacity to irrigate or suction the small intestine to remove bile, chyme, or air bubbles that may obscure visualization. However, the optimal bowel preparation required before CE remains unknown and is an active area of endoscopy research.

Study Rationale

Like colonoscopy, research in bowel preparation for CE has focused on the use of oral purgatives such as polyethylene glycol (PEG) to cleanse the gastrointestinal tract.([21-29](#_ENREF_21)) However, bowel preparation for CE has the unique requirement of also having to remove air bubbles from the lumen. Due to its inability to irrigate or suction, air bubbles left in the small bowel can significantly obstruct visualization of the underlying mucosa during CE (Figure 1).([30](#_ENREF_30)) Unfortunately, osmotic laxatives such as PEG do not remove air bubbles. Instead, non-absorbable simethicone (Ovol^®^, Church & Dwight) is used to reduce the surface tension of air bubbles leading to their collapse and dispersion.

Simethicone has been used with some success in past clinical trials (Table 1). Two studies compared PEG alone to PEG combined with simethicone and found superior visualization quality with the latter.([31](#_ENREF_31), [32](#_ENREF_32)) Albert et al.([33](#_ENREF_33)) compared simethicone with fasting overnight and also noted an improvement in visualization quality with simethicone. However, more recent studies have reported improvement in visualization quality only in the proximal half of the small intestine ([34](#_ENREF_34), [35](#_ENREF_35)) or no overall improvement.([36](#_ENREF_36), [37](#_ENREF_37)) Direct comparison between these studies is problematic due to differences in simethicone dosage, use of PEG, and outcome measures which usually lacked validation. Within these limitations, two meta-analysis have been performed with results favoring the use of simethicone for this indication.([38](#_ENREF_38), [39](#_ENREF_39)) Thus, the totality of evidence is in favour of simethicone although the dosage required to achieve consistent results is unknown.

One potential explanation for inconsistent cleansing with simethicone in current practice is that an insufficient volume is being used. During CE, simethicone is mixed in water to create a ‘cleaning’ solution and past studies have used volumes ranging between 0.5 ml to 200 ml, often at a concentration of 1.5mg/ml.([31-37](#_ENREF_31), [40](#_ENREF_40)) Given the size of the stomach, where the simethicone solution pools and becomes diluted with gastric juices, and the length of the small intestine, where the simethicone solution is further diluted, we hypothesize that a larger volume of simethicone solution would produce more consistent cleansing of the small intestine. This notion is supported by two studies that demonstrated improved visualization limited to the proximal half but no difference in the distal half of the small bowel, possibly due to insufficient simethicone solution reaching distally.([34](#_ENREF_34), [35](#_ENREF_35))

Pilot Data

We recently examined this hypothesis in a small pilot study (NCT#02334631) where 30 participants were randomized to high volume simethicone (750 ml at 1.5 mg/ml) or standard volume simethicone (200 ml at 1.5 mg/ml). We chose to use an active comparator rather than placebo as we felt the totality of evidence was in support of giving some simethicone although the optimal amount is yet unknown.([38](#_ENREF_38)) We chose 750 ml for the experimental arm as a compromise between palatability and maximizing the volume that can be delivered into the small bowel. For our control arm, we used 200 ml as was standard practice at our centre. Both solutions were prepared as 1.5 mg/ml.

Visualization quality in our study was assessed blindly using a validated scale developed by Park et al.([41](#_ENREF_41)). The Park score involves assessing the first frame of every 5 minute interval in the CE video using a 4 step scale for visualized mucosa and degree of obstruction to calculate a visualization quality score (Table 2). In deriving the score, Park validated it against an extremely labour intensive scale where every frame within the first 2 minutes of every 5 minute interval was manually assessed. During validation, the Park score had excellent agreement with the labour intensive scale (intraclass correlation coefficient, ICC = 0.82) and interobserver agreement between three readers (ICC = 0.80) and intraobserver agreement 4 weeks later (ICC = 0.76) were good to excellent. Based on the receiver operating characteristics curve, the authors defined adequate bowel preparation as a Park Score ≥ 2.2.

Using this scale, our pilot study demonstrated a strong trend towards significant improvement in bowel preparation adequacy (87% versus 53%, p=0.05) and visualization quality scores (2.44 versus 2.23, p=0.13) among those given high volume simethicone (Table 3, 4). Statistical significance was not reached in this small pilot study limited to 30 participants. However, in keeping with our hypothesis, improvements in scores were noted in both the proximal and distal half of the small intestine.

The second objective of the pilot study was to determine the feasibility of our study protocol (Figure 2). As expected, there were no drop outs (since subject participation is limited to the day of CE), no adverse events (since simethicone is an over-the-counter non-absorbable anti-flatulent with no contraindication or adverse events noted in the Compendium of Pharmaceuticals and Specialties), and no recruitment obstacles (due to the use of an active control, safety profile of the experimental intervention, and no requirement for study visits). Furthermore, there were no issues with palatability and all subjects were able to ingest the amount they were randomized to (ie. 100% adherence to randomized regimen).

Proposed Study

As a follow up to the encouraging data found in our recently completed pilot study, we now wish to conduct a double blind randomized clinical trial to compare high volume simethicone (750 ml at 1.5 mg/ml) and standard volume simethicone (200 ml at 1.5 mg/ml) in achieving adequate bowel preparation as defined by a validated scale ([41](#_ENREF_41)) for CE. Based on our pilot data, we hypothesize high volume simethicone will better cleanse the small intestine leading to a higher bowel preparation adequacy rate and result in more small bowel diagnoses.

**STATEMENT OF OBJECTIVES & SPECIFIC AIMS**

Specific Aim #1:

To compare the CE bowel preparation adequacy rate, as defined by Park et al.([41](#_ENREF_41)), between high volume and standard volume simethicone. Based on our pilot study, we hypothesize a greater proportion of subjects will achieve adequate bowel preparation with high volume than standard volume simethicone.

Specific Aim #2:

To compare the Park score, stratified by segments (proximal half, distal half, total small bowel), between high volume and standard volume simethicone. Based on our pilot study, we hypothesize the mean score for each segment will be higher for high volume compare to standard volume simethicone.

Specific Aim #3:

To compare the CE diagnostic rate between high volume and standard volume simethicone. We hypothesize high volume simethicone will lead to a higher diagnostic rate than standard volume due to superior cleansing.

**STATEMENT OF RELEVANCE FOR ONTARIANS**

Our study has the potential to positively impact Ontarians in two ways. First, it may have a direct benefit for patients undergoing CE in this province by improving visualization quality. Although considered to be the gold standard in imaging of the small intestine, rivaled only by intraoperative enteroscopy which carries a mortality risk, CE is diagnostic in only 50-60% of cases.([19](#_ENREF_19)) Thus, improvement in diagnostic rates for this procedure is needed and it is our hope that improved bowel preparation quality will translate into more diagnoses for Ontarians. However, weather this is achievable is unknown and is one of the goals of this study.

Second, a positive study may save valuable health care resources and improve access to this procedure. Due to the cost of the single use disposable capsule (ie. >$600/unit), the availability of this procedure is tightly regulated by the provincial government and only a limited number are funded each year. As an example, our CE program is the only one serving Southwestern Ontario and receives more than 200 referrals a year. Unfortunately, our program is only funded to perform 100 CE annually, resulting in long wait times (~9 months) and many patients being denied the procedure. In the setting of limited resources, every capsule counts. However, those with inadequate bowel preparation must repeat the procedure, resulting in one less capsule available for other Ontarians. If our study hypothesis proves to be correct, there will be fewer patients with inadequate bowel preparation, which would translate into improved access province wide once our findings are published.

**METHODOLOGY**

Study Design & Duration

This is a double blind randomized clinical trial evaluating high volume simethicone (750 ml at 1.5 mg/ml) versus standard volume simethicone (200 ml at 1.5 mg/ml) for CE. The study will be performed at London Health Sciences Center-Victoria Hospital with a recruitment period of 24 months calculated as follows: 100 CE performed annually based on our approved funding model from the province, a conservative 80% recruitment rate based on the pilot study, 0% drop out since the study is completed on the day of enrollment, and a sample size of 164 based on the sample size calculation below. Another 6 months will be required to analyze our results and prepare for publication. Thus, we anticipate the entire study will take 2.5 years to complete.

Randomization & Blinding

Randomization will be performed in blocks of varying sizes through a web based interface, Research Electronic Data Capture (REDCap), to ensure concealed allocation. Patients, physicians, and outcome assessors will be blinded to randomization allocation.

Patient blinding is particularly challenging since the two treatment arms involve ingesting a different volume of solution (ie. 200 ml vs. 750 ml). This creates an opportunity for unmasking as patients may be able to deduce which treatment arm they were randomized to based on the weight of the cup and the amount ingested. Furthermore, water cannot be used to equalize the volume between the two treatment arms due to dilution of the standard volume solution if water is added to it. In addition, inert weights cannot be added to the standard volume solution since the patient may still be able to deduce treatment allocation based on the amount ingested. To prevent unmasking, identical non-transparent lidded cups sealed with tamper tape will be used and patients will not be informed of the volumes being compared. Thus, even if patients are able to estimate the solution volume based on weight and amount ingested, they will still be blinded to randomization results since they would not know the volumes that define the standard and high volume groups. To meet the requirements of our REB, patients would be informed beforehand that they may be asked to drink between 1 to 900 ml of solution. In addition, a letter will be given to each patient after study completion informing them of the volumes in the two treatment arms and their randomization results.

Study Population

All patients over 18 years of age scheduled to undergo a CE of the small bowel for any indication at London Health Sciences Centre will be considered for recruitment.

Inclusion Criteria

1. Any patient undergoing small bowel CE for any indication

Exclusion Criteria

1. Patients with a contraindication to CE (GI tract stricture, oropharyngeal dysphagia, pregnancy)
2. Endoscopic insertion of the capsule
3. Inpatient procedures for active GI bleeding
4. Patients with fluid restrictions or those who believe they would be unable to drink up to 900 ml of fluid within 10 minutes prior to the VCE

Intervention

All patients will take a standard small bowel preparation consisting of the following starting the day before the procedure: clear fluids starting at lunch, two liters of PEG at 8 PM, and fasting starting at midnight. Patients arrive in the endoscopy unit at 6:45 AM the following morning. Randomization is performed via REDCap and simethicone given as follows: those randomized to high volume simethicone will drink a 750 ml solution (1125 mg simethicone diluted in 750 ml of water, 1.5 mg/ml) and those randomized to standard volume simethicone will drink a 200 ml solution (300 mg simethicone diluted in 200 ml of water, 1.5 mg/ml) in identical non-transparent lidded cups sealed with tamper tape prepared by the hospital’s Clinical Trials Pharmacy and administered to the patient by the research nurse. Regardless of randomization results, the solution will be consumed over a 5 minute period and the patient instructed to resume fasting for 30 minutes before swallowing the capsule. Adherence will be assessed by collection of the cups after ingestion.

After swallowing the capsule, the patient is discharged from the endoscopy unit, encouraged to remain active for the day, allowed to have clear fluids in 2 hours, a light meal in 4 hours, and to return the CE recorder to the endoscopy unit at 5 PM. Once the CE recorder is returned, the patient is discharged from the endoscopy unit and has completed the study. Subjects will be called by the study nurse 7 days later to assess for adverse events.

CE will be performed with the Given Imaging SB3 capsule and reviewed with the accompanying software, RAPID v8.3. Videos will be reviewed in a blinded fashion without knowledge of randomization results using the Park score ([41](#_ENREF_41)) as follows:

1. First duodenal and first cecal image are marked. The interval between these two landmarks defines the small bowel transit time.
2. The small bowel transit time is manually divided into 5 minute intervals and the first image of every 5 minute interval is selected. For example, if the first duodenal image occurred at 13 minute 52 second, the next image is selected at 18 minute 52 seconds followed by 23 minute 52 seconds and so forth until the cecum is reached.
3. Each selected image is given a visualized mucosa score and degree of obstruction score as follows:
   1. Visualized Mucosa Score (VMS)
      1. 3 points: >75% of mucosa is seen
      2. 2 points: 50-75% of mucosa is seen
      3. 1 point: 25-49% of mucosa is seen
      4. 0 point: <25% of mucosa is seen
   2. Degree of Obstruction Score (DOS)
      1. 3 points: <5% of view is obstructed
      2. 2 points: 5-25% of view is obstructed
      3. 1 point: 26-50% of view is obstructed
      4. 0 point: >50% of view is obstructed
4. The visualization quality score (VQS) is calculated as follows:
   1. VQS = (mean VMS + mean DOS) / 2
5. The VMS, DOS, and VQS will be assessed based on the following segments:
   1. Total small intestine (0-100% SI transit time)
   2. Proximal half of the small intestine (0-49% SI transit time)
   3. Distal half of the small intestine (50-100% SI transit time)

Baseline Data Collection

The following information will be collected at the time of enrollment: hospital ID number, age, sex, indication, previous relevant investigations (i.e upper endoscopy, colonoscopy, small bowel imaging), and risk factors for intestinal dysmotility (history of diabetes, scleroderma, or medications including anticholinergics and opioids).

Outcome Measures

*Primary outcome*

1. Adequate bowel preparation defined as VQS > 2.25 ([41](#_ENREF_41))

*Secondary outcomes*

1. Mean Park score
2. Diagnostic yield
   1. Any of the following lesions will be considered diagnostic: angioectasia, polyp, tumor/mass, ulcer, Crohn’s disease, stricture
   2. Note that small non-specific red spots of questionable significance will not be considered diagnostic
3. Study completion rate
   1. Defined as the capsule reaching the cecum
4. Gastric emptying time
   1. Defined as the time interval between the first gastric and first duodenal image
5. Small intestinal transit time
   1. Defined as the time interval between the first duodenal and first cecal image

Adverse Events

Since simethicone is a non-absorbable over-the-counter medication, we do not anticipate any adverse events. In addition, there are no contraindications or adverse events noted in the Compendium of Pharmaceuticals and Specialties nor were there any seen in our pilot study. However, any possible adverse events will be assessed at the time of the procedure and through a phone call 7 days later. Minor adverse events will be defined as: any new (or worsening if chronic) abdominal pain, bloating, flatulence, constipation, diarrhea, nausea. Serious adverse events will be defined as any symptom resulting in an emergency room visit, hospitalization, or death.

Regulatory Approval

We have already secured regulatory approval and a No Objection Letter from Health Canada (control #190759) and our local Research Ethics Board (HSREB #106269). We recently submitted amendments based on a revised sample size and the amendments are currently under review. However, we do not anticipate any regulatory barriers should this grant application be funded.

Statistical Analysis

Baseline demographic data following a normal distribution will be reported as mean and standard deviation (SD) while non-parametric data will be reported as median and range. Categorical data will be presented as proportions. Differences in continuous outcomes will be analyzed with t-test (if normally distributed) and Wilcoxon rank sum test (for non-parametric data). Differences in categorical outcomes will be analyzed with Chi-square test or Fisher’s exact test as deemed appropriate. All data will be analyzed as intention to treat.

The sample size was calculated using conservative estimates from our pilot study as follows: assuming 80% adequacy rate in the experimental arm (87% in pilot study), 60% in the control arm (53% in pilot study), a two sided significance level of 0.05, and power of 0.80, 164 participants are needed in the study.

Study Strengths & Limitations

The primary strength of this study is its rigorous design. Through the use of randomization, double blinding, and intention to treat analyzes, we can minimize confounding and bias in our study. Since our intervention is simple, inexpensive, and well tolerated, positive findings can easily improve clinical practice given the low frequency with which adequate small bowel preparation is achieved with standard volume simethicone (ie. 47% in our pilot study using the rigorous Park criteria). We anticipate that this study will be of sufficient quality to be included in future guidelines concerning CE.

Mentorship is another strength of this grant proposal. Although Dr. Sey is a young investigator, he has completed a clinical scholar year in research methods at Harvard Medical School with a concentration in clinical trials and is currently working on his Master of Public Health at Harvard University. More importantly, he is mentored closely by Dr. Brian Feagan. Dr. Feagan is an experienced clinical trialist with an established publication record. Although Dr. Feagan’s main field is in inflammatory bowel disease, he has also worked in gastrointestinal imaging and endoscopy in particular. Dr. Sey meets with Dr. Feagan on a regular basis and minutes are kept and reported to the Department of Medicine.

The primary limitation of our study is its single centre design, which limits the sample size for feasibility reasons and perhaps generalizability due to the practice pattern of a single hospital. However, a multi-centre study would be orders of magnitude more expensive than the current grant proposal. Furthermore, multi-centre RCT in the field of CE are rare. Despite being a single centre study, our sample size is sufficient to detect a meaningful difference in bowel preparation adequacy. Accordingly, we believe a single centre design is the most efficient way of answering this study question.

**BUDGET**

| **ITEM** | **Year 1** | **Year 2** | **Year 3** | **Total** |
| --- | --- | --- | --- | --- |
| Simethicone | $2,967 | $2,967 | - | $5,234 |
| PegLyte | $2,460 | $2,460 |  | $4,860 |
| Research Nurse | $7,996 | $7,996 | $3,998 | $19,990 |
| Summer Research Student | $5,000 | - | - | $5,000 |
| Biostatistician support | $650 | - | $1,950 | $2,300 |
| Conference cost | - | - | $1,500 | $1,500 |
| Printing cost | $115 | - | - | $115 |
| Long term storage cost | - | - | $500 | $500 |
| REB cost | - | - | - | $0 |
| Overhead | - | - | - | $0 |
| Research & Office space | - | - | - | $0 |
| **TOTAL** | $19,188 | $13,423 | $7,948 | $40,559 |

Please note: Funds for years 1 & 2 are to support subject recruitment and data collection (total = 24 months). Funds for year 3 is to support 6 months of data clean up, analysis, study presentation, publication, and closeout.

Justification of Expenses

- Simethicone
  - Cost estimate prepared by London Health Sciences Centre Pharmacy Department – Clinical Trials Service
  - Includes all costs associated with preparation of the simethicone solution at the requested concentrations in study containers sealed with tamper tape, delivery, dispensing fees, storage fees, record keeping, HST, etc…for the duration of subject recruitment (ie. years 1 and 2)
- PegLyte
  - $30/package of PegLyte x 164 patients = $4,920
- REDCap
  - Research Electronic Data Capture used for online randomization and data entry
  - $100USD /month via Lawson Health Research Institute x 24 months of recruitment = $3,122 CAD
- Research Nurse
  - Research nurse to assist with identification of potential subjects, obtaining informed consent, study enrollment, randomization, administration of simethicone solution, data collection and entry, follow up phone calls for adverse events, paperwork related to REB, local hospital Clinical Research Impact Committee, and Health Canada. I anticipate this will require 4 hours of work/week.
  - Based on the payroll for our current Inflammatory Bowel Disease Research Nurse: $31.00/hour + 6% vacation pay + 9% in lieu of benefits + 9% pension contribution x 4 hours/week x 130 weeks = $19,989.
- Summer Research Student
  - To provide a stipend for a summer undergraduate research student (ie. in either the health sciences or medical/nursing school) to work with Dr. Sey for 16 weeks during the first year of the study
  - The student’s involvement would include assisting with subject enrolment, consent, data entry, and data analysis and synthesis
  - The student would join other summer research students in a biweekly lunch and learn session with Dr. Sey to discuss research methods and epidemiology
  - The student would also be involved in other studies time permitting although this would be his/her primary project
  - The fixed stipend would be for $5,000 for 16 weeks of work over the summer
- Biostatistician Support
  - $130/hour (based on discounted cost for Western University affiliated faculty) x 20 hours = $2,600
  - 5 hours will be dedicated to year 1 for statistical consultation prior to study initiation and 15 hours dedicated to year 3 after completion of data collection for final data clean up, analysis, and consultation
- Conference cost
  - Cost associated with flight & accommodations to present at Digestive Disease Week held annually in the USA
  - $1,500
- Printing costs
  - Printing costs for 164 consent forms (5 pages) in duplicates at 7 cents/page
  - $115
- Long term storage costs
  - Cost associated with storage of data for 25 years as mandated by Health Canada and final data destruction (paper and electronic)
  - $500
- REB costs
  - There is no REB fees since this is an investigator sponsored/initiated study
- Overhead fee
  - There is no overhead fees since this is an investigator sponsored /initiated study
- Research & Office space
  - There is no cost since this is already provided by the London Health Sciences Centre-Victoria Campus to Dr. Sey as a condition of his appointment as a clinician-researcher at Western University

**REFERENCES**

1. Committee AT, Wang A, Banerjee S, Barth BA, Bhat YM, Chauhan S, et al. Wireless capsule endoscopy. Gastrointestinal endoscopy. 2013;78(6):805-15.

2. Moglia A, Pietrabissa A, Cuschieri A. Capsule endoscopy. Bmj. 2009;339:b3420.

3. Ladas SD, Triantafyllou K, Spada C, Riccioni ME, Rey JF, Niv Y, et al. European Society of Gastrointestinal Endoscopy (ESGE): recommendations (2009) on clinical use of video capsule endoscopy to investigate small-bowel, esophageal and colonic diseases. Endoscopy. 2010;42(3):220-7.

4. Committee ASoP, Fisher L, Lee Krinsky M, Anderson MA, Appalaneni V, Banerjee S, et al. The role of endoscopy in the management of obscure GI bleeding. Gastrointestinal endoscopy. 2010;72(3):471-9.

5. Iddan G, Meron G, Glukhovsky A, Swain P. Wireless capsule endoscopy. Nature. 2000;405(6785):417.

6. Pennazio M, Spada C, Eliakim R, Keuchel M, May A, Mulder CJ, et al. Small-bowel capsule endoscopy and device-assisted enteroscopy for diagnosis and treatment of small-bowel disorders: European Society of Gastrointestinal Endoscopy (ESGE) Clinical Guideline. Endoscopy. 2015;47(4):352-76.

7. Sami SS, Al-Araji SA, Ragunath K. Review article: gastrointestinal angiodysplasia - pathogenesis, diagnosis and management. Alimentary pharmacology & therapeutics. 2014;39(1):15-34.

8. Wang Z, Chen JQ, Liu JL, Qin XG, Huang Y. CT enterography in obscure gastrointestinal bleeding: a systematic review and meta-analysis. Journal of medical imaging and radiation oncology. 2013;57(3):263-73.

9. Wiarda BM, Heine DG, Mensink P, Stolk M, Dees J, Hazenberg HJ, et al. Comparison of magnetic resonance enteroclysis and capsule endoscopy with balloon-assisted enteroscopy in patients with obscure gastrointestinal bleeding. Endoscopy. 2012;44(7):668-73.

10. Barret M, Malamut G, Rahmi G, Samaha E, Edery J, Verkarre V, et al. Diagnostic yield of capsule endoscopy in refractory celiac disease. The American journal of gastroenterology. 2012;107(10):1546-53.

11. Teshima CW, Kuipers EJ, van Zanten SV, Mensink PB. Double balloon enteroscopy and capsule endoscopy for obscure gastrointestinal bleeding: an updated meta-analysis. Journal of gastroenterology and hepatology. 2011;26(5):796-801.

12. Jensen MD, Nathan T, Rafaelsen SR, Kjeldsen J. Diagnostic accuracy of capsule endoscopy for small bowel Crohn's disease is superior to that of MR enterography or CT enterography. Clinical gastroenterology and hepatology : the official clinical practice journal of the American Gastroenterological Association. 2011;9(2):124-9.

13. Dionisio PM, Gurudu SR, Leighton JA, Leontiadis GI, Fleischer DE, Hara AK, et al. Capsule endoscopy has a significantly higher diagnostic yield in patients with suspected and established small-bowel Crohn's disease: a meta-analysis. The American journal of gastroenterology. 2010;105(6):1240-8; quiz 9.

14. Cheung DY, Lee IS, Chang DK, Kim JO, Cheon JH, Jang BI, et al. Capsule endoscopy in small bowel tumors: a multicenter Korean study. Journal of gastroenterology and hepatology. 2010;25(6):1079-86.

15. Rondonotti E, Pennazio M, Toth E, Menchen P, Riccioni ME, De Palma GD, et al. Small-bowel neoplasms in patients undergoing video capsule endoscopy: a multicenter European study. Endoscopy. 2008;40(6):488-95.

16. Golder SK, Schreyer AG, Endlicher E, Feuerbach S, Scholmerich J, Kullmann F, et al. Comparison of capsule endoscopy and magnetic resonance (MR) enteroclysis in suspected small bowel disease. International journal of colorectal disease. 2006;21(2):97-104.

17. Cobrin GM, Pittman RH, Lewis BS. Increased diagnostic yield of small bowel tumors with capsule endoscopy. Cancer. 2006;107(1):22-7.

18. Bailey AA, Debinski HS, Appleyard MN, Remedios ML, Hooper JE, Walsh AJ, et al. Diagnosis and outcome of small bowel tumors found by capsule endoscopy: a three-center Australian experience. The American journal of gastroenterology. 2006;101(10):2237-43.

19. Triester SL, Leighton JA, Leontiadis GI, Fleischer DE, Hara AK, Heigh RI, et al. A meta-analysis of the yield of capsule endoscopy compared to other diagnostic modalities in patients with obscure gastrointestinal bleeding. The American journal of gastroenterology. 2005;100(11):2407-18.

20. Hartmann D, Schmidt H, Schilling D, Kinze F, Eickhoff A, Weickert U, et al. Follow-up of patients with obscure gastrointestinal bleeding after capsule endoscopy and intraoperative enteroscopy. Hepato-gastroenterology. 2007;54(75):780-3.

21. Magalhaes-Costa P, Carmo J, Bispo M, Santos S, Chagas C. Superiority of the Split-dose PEG Regimen for Small-Bowel Capsule Endoscopy: A Randomized Controlled Trial. Journal of clinical gastroenterology. 2015.

22. Ito T, Ohata K, Ono A, Chiba H, Tsuji Y, Sato H, et al. Prospective controlled study on the effects of polyethylene glycol in capsule endoscopy. World journal of gastroenterology : WJG. 2012;18(15):1789-92.

23. Belsey J, Crosta C, Epstein O, Fischbach W, Layer P, Parente F, et al. Meta-analysis: efficacy of small bowel preparation for small bowel video capsule endoscopy. Current medical research and opinion. 2012;28(12):1883-90.

24. Pons Beltran V, Gonzalez Suarez B, Gonzalez Asanza C, Perez-Cuadrado E, Fernandez Diez S, Fernandez-Urien I, et al. Evaluation of different bowel preparations for small bowel capsule endoscopy: a prospective, randomized, controlled study. Digestive diseases and sciences. 2011;56(10):2900-5.

25. Park SC, Keum B, Seo YS, Kim YS, Jeen YT, Chun HJ, et al. Effect of bowel preparation with polyethylene glycol on quality of capsule endoscopy. Digestive diseases and sciences. 2011;56(6):1769-75.

26. Lapalus MG, Ben Soussan E, Saurin JC, Favre O, D'Halluin PN, Coumaros D, et al. Capsule endoscopy and bowel preparation with oral sodium phosphate: a prospective randomized controlled trial. Gastrointestinal endoscopy. 2008;67(7):1091-6.

27. Franke A, Hummel F, Knebel P, Antoni C, Bocker U, Singer MV, et al. Prospective evaluation of small bowel preparation with bisacodyl and sodium phosphate for capsule endoscopy. World journal of gastroenterology : WJG. 2008;14(13):2061-4.

28. van Tuyl SA, den Ouden H, Stolk MF, Kuipers EJ. Optimal preparation for video capsule endoscopy: a prospective, randomized, single-blind study. Endoscopy. 2007;39(12):1037-40.

29. Viazis N, Sgouros S, Papaxoinis K, Vlachogiannakos J, Bergele C, Sklavos P, et al. Bowel preparation increases the diagnostic yield of capsule endoscopy: a prospective, randomized, controlled study. Gastrointestinal endoscopy. 2004;60(4):534-8.

30. Song HJ, Moon JS, Do JH, Cha IH, Yang CH, Choi MG, et al. Guidelines for Bowel Preparation before Video Capsule Endoscopy. Clinical endoscopy. 2013;46(2):147-54.

31. Fang YH, Chen CX, Zhang BL. Effect of small bowel preparation with simethicone on capsule endoscopy. Journal of Zhejiang University Science B. 2009;10(1):46-51.

32. Wei W, Ge ZZ, Lu H, Gao YJ, Hu YB, Xiao SD. Purgative bowel cleansing combined with simethicone improves capsule endoscopy imaging. The American journal of gastroenterology. 2008;103(1):77-82.

33. Albert J, Gobel CM, Lesske J, Lotterer E, Nietsch H, Fleig WE. Simethicone for small bowel preparation for capsule endoscopy: a systematic, single-blinded, controlled study. Gastrointestinal endoscopy. 2004;59(4):487-91.

34. Chen HB, Huang Y, Chen SY, Song HW, Li XL, Dai DL, et al. Small bowel preparations for capsule endoscopy with mannitol and simethicone: a prospective, randomized, clinical trial. Journal of clinical gastroenterology. 2011;45(4):337-41.

35. Ge ZZ, Chen HY, Gao YJ, Hu YB, Xiao SD. The role of simeticone in small-bowel preparation for capsule endoscopy. Endoscopy. 2006;38(8):836-40.

36. Rosa BJ, Barbosa M, Magalhaes J, Rebelo A, Moreira MJ, Cotter J. Oral purgative and simethicone before small bowel capsule endoscopy. World journal of gastrointestinal endoscopy. 2013;5(2):67-73.

37. Spada C, Riccioni ME, Familiari P, Spera G, Pirozzi GA, Marchese M, et al. Polyethylene glycol plus simethicone in small-bowel preparation for capsule endoscopy. Digestive and liver disease : official journal of the Italian Society of Gastroenterology and the Italian Association for the Study of the Liver. 2010;42(5):365-70.

38. Kotwal VS, Attar BM, Gupta S, Agarwal R. Should bowel preparation, antifoaming agents, or prokinetics be used before video capsule endoscopy? A systematic review and meta-analysis. European journal of gastroenterology & hepatology. 2014;26(2):137-45.

39. Wu L, Cao Y, Liao C, Huang J, Gao F. Systematic review and meta-analysis of randomized controlled trials of Simethicone for gastrointestinal endoscopic visibility. Scandinavian journal of gastroenterology. 2011;46(2):227-35.

40. Postgate A, Tekkis P, Patterson N, Fitzpatrick A, Bassett P, Fraser C. Are bowel purgatives and prokinetics useful for small-bowel capsule endoscopy? A prospective randomized controlled study. Gastrointestinal endoscopy. 2009;69(6):1120-8.

41. Park SC, Keum B, Hyun JJ, Seo YS, Kim YS, Jeen YT, et al. A novel cleansing score system for capsule endoscopy. World journal of gastroenterology : WJG. 2010;16(7):875-80.

A B


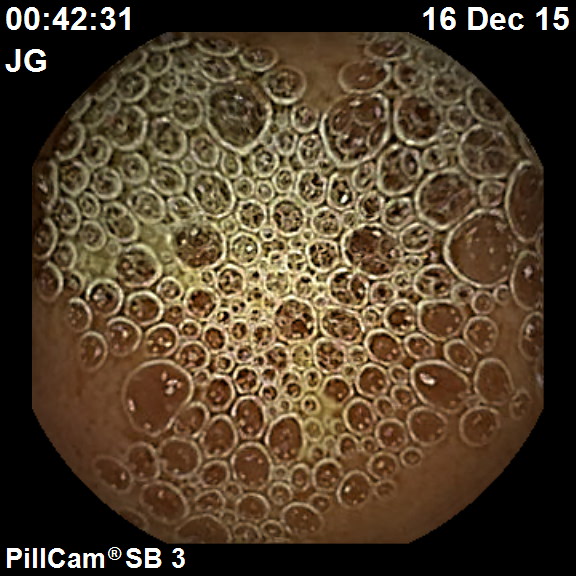

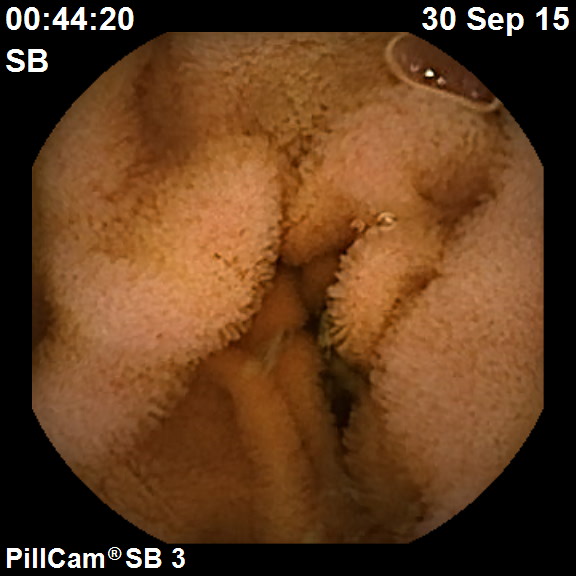


**Figure 1:** Example of poor visualization of the small intestine due to air bubbles (A) and good visualization with the use of simethicone (B)


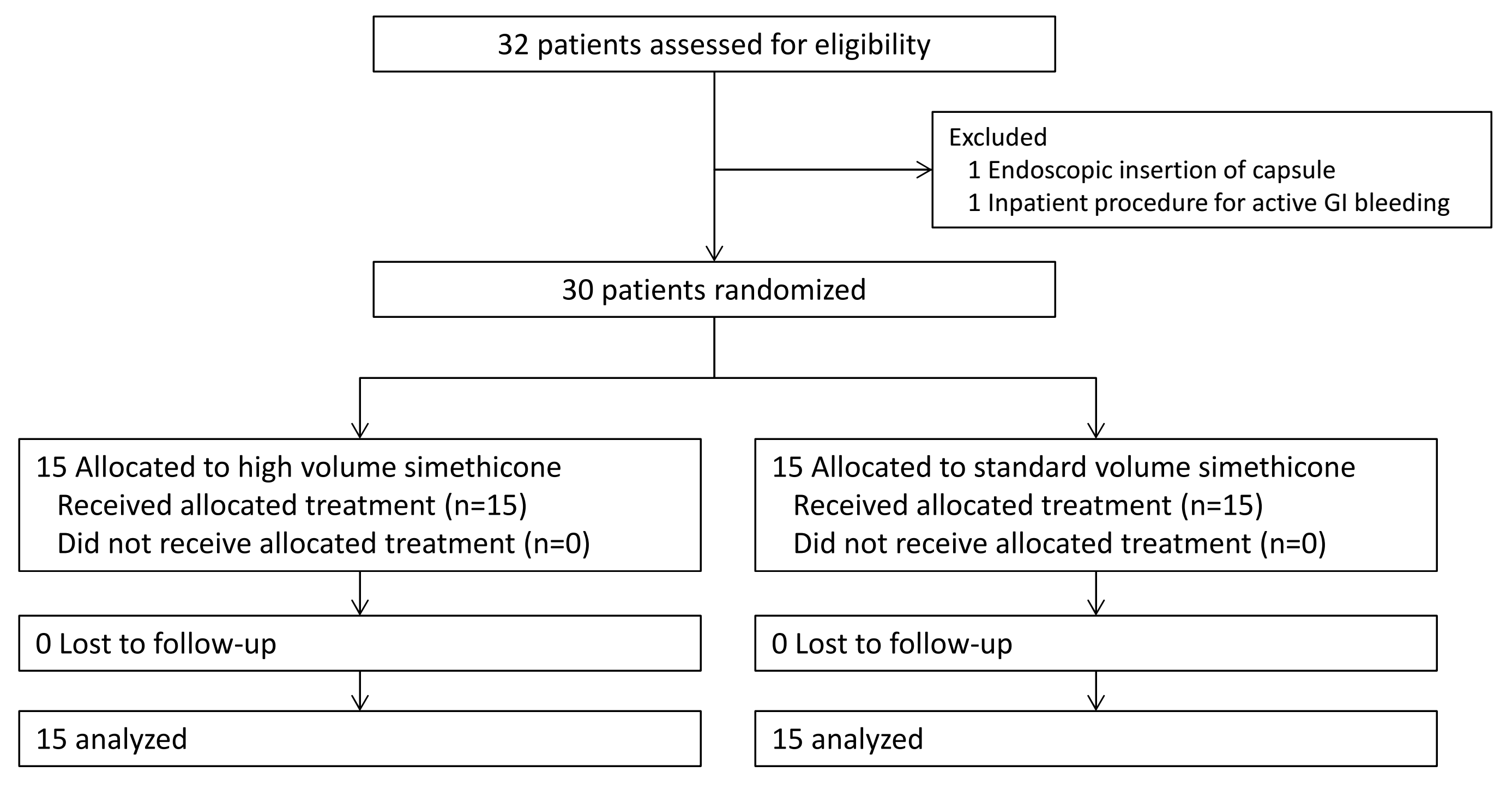
**Figure 2:** Enrolment profile of pilot study


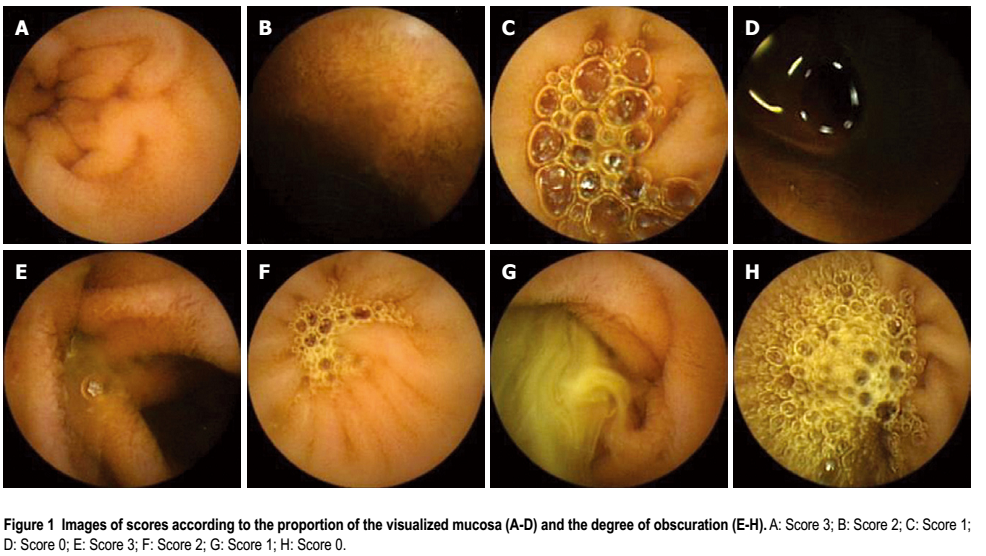


| **Visualized Mucosa Score (VMS)**  >75%  50-75%  25-49%  <25% | **Score**  3  2  1  0 |
| --- | --- |
| **Degree of Obstruction Score (DOS)**  <5%  5-25%  26-50%  >50% | **Score**  3  2  1  0 |
| **Visualization Quality Score** | (Mean VMS + Mean DOS)/2 |

**Table 2:** Park Score ([41](#_ENREF_41))

|  | **Standard Volume Simethicone**  **(n = 15 )** | **High Volume**  **Simethicone**  **(n = 15)** |
| --- | --- | --- |
| Age-mean (SD) | 60.8 (15.7) | 65.5 (19.4) |
| Female-no. (%) | 6 (40%) | 7 (47%) |
| Indication-no. (%)  Obscure overt gastrointestinal bleeding  Obscure occult gastrointestinal bleeding  Crohn’s disease  Small bowel mass | 6 (40%)  7 (47%)  1 (7%)  1 (7%) | 5 (33%)  7 (47%)  -  3 (20%) |
| Risk factors for intestinal dysmotility-no. (%)  Narcotics  Anti-cholinergic  Calcium channel blocker  Diabetes  Primary autonomic dysfunction | 4 (27%)  2  1  3  -  - | 9 (60%)  -  -  3  5  1 |
| Prior tests of the small intestine-no. (%)  Capsule endoscopy  SBFT  CT abdomen  MR enterography  Small bowel ultrasound  Meckel’s scan | 4 (27%)  2 (13%)  -  4 (27%)  -  1 (7%)  1 (7%) | 4 (27%)  1 (7%)  -  4 (27%)  2 (13%)  -  - |

**Table 3:** Pilot study baseline demographics

|  | **Standard Volume Simethicone**  **(n = 15)** | **High Volume**  **Simethicone**  **(n = 15)** | **p-value** |
| --- | --- | --- | --- |
| Gastric transit time-mean (SD) | 21.9 (30.8) | 40.9 (31.8) | 0.11 |
| Small intestine transit time-mean (SD) | 251.0 (81.5) | 214.9 (119.3) | 0.34 |
| Incomplete study-no (%)^a^ | 0 (0%) | 1 (7%) | 0.31 |
| Visualized Mucosa Score-mean (SD)  Proximal half  Distal half  Entire small intestine | 2.53 (0.37)  2.28 (0.53)  2.45 (0.37) | 2.69 (0.33)  2.48 (0.24)  2.64 (0.18) | 0.21  0.19  0.08 |
| Degree of Obstruction Score-mean (SD)  Proximal half  Distal half  Entire small intestine | 2.17 (0.40)  1.90 (0.59)  2.09 (0.39) | 2.29 (0.48)  2.07 (0.36)  2.24 (0.32) | 0.47  0.34  0.25 |
| Visualization Quality Score-mean (SD)  Proximal half  Distal half  Entire small intestine | 2.35 (0.37)  2.09 (0.56)  2.23 (0.45) | 2.49 (0.40)  2.28 (0.27)  2.44 (0.24) | 0.32  0.25  0.13 |
| Adequate bowel preparation^b^ | 8 (53%) | 13 (87%) | 0.05 |

^a^Denotes failure to reach the cecum prior to the end of the study

^b^Defined as visualization quality score for the entire small intestine ≥ 2.25

**Table 4:** Pilot study results
